# Korea's Response to COVID-19 According to Set Time Frames, With a Focus on the Network Between the Government and Responding Agencies: Social Network Analysis

**DOI:** 10.2196/35958

**Published:** 2022-05-23

**Authors:** Jungyun Cho, Wook Kang, Julak Lee

**Affiliations:** 1 Department of Public Administration Korean National Police University Asan Republic of Korea; 2 Department of Industrial Security Chung-Ang University Seoul Republic of Korea

**Keywords:** COVID-19, government departments’ networks, network structure, contagious disease response, social network analysis

## Abstract

**Background:**

In December 2019, COVID-19 was first confirmed in Wuhan, China, and as the respiratory disease spread around the globe, there was a spike in interest worldwide in combating such contagious diseases. When such disasters occur, the central government of South Korea and its affiliated local governments—together with nongovernmental organizations—play a crucial role in crisis management systems.

**Objective:**

The purpose of this paper is to corroborate the characteristics government ministries and domestic and foreign institutions exhibit through their interconnection when the parties are undergoing a disease-related catastrophe such as the COVID-19 pandemic.

**Methods:**

Using the social network analysis technique, the span of the COVID-19 pandemic was segmented into 3 time frames, and the relational characteristics of the COVID-19 contagious disease response department and related agencies at home and abroad were analyzed based on 3 centralities.

**Results:**

Evidence from the second and third time frames indicates that the agents reacting to contagious diseases do not necessarily hold the central position in the network. From this, it can be inferred that it is not only the primary host that plays a pivotal role but the key to a successful response to various disasters also lies in cooperation with the relevant parties.

**Conclusions:**

The incongruency between the findings of this paper and the existing disaster response system gives rise to the corollary that both the essential parties and the adjoining ones need to collaborate for a coordinated crisis response in disaster situations. Furthermore, much significance lies in the fact that this paper explores the various aspects that could surface among the host and relevant parties in a real-life pandemic.

## Introduction

### Background

In December 2019, COVID-19 (a respiratory disease informally known as coronavirus) originated in Wuhan, China [1-3]. Since then, interest in responding to contagious diseases has increased worldwide as it spread across China and around the globe [4].

With the first contraction of the disease on South Korean territory being reported on January, 20, 2020, an exponential growth of cases occurred, with the largest number of infections traced back to Daegu; a local blockade was seriously considered [5]. In the event of such a disaster, the foremost mission of the standing government is to protect its citizens from harm, which is why the crisis management system operates for the safety of the people. Perry [6] stated that local, state, federal, and private organizations play a central role in a crisis management system. Putting this into the context of South Korea, this translates to the central government, local governments, private organizations, and nongovernmental organizations (NGOs), and in defense of the rapid transmission of COVID-19, the nation has ensured communication with the Infectious Disease Response Center and its affiliated departments for assured support and cooperation.

Social network analysis (SNA) has been applied to understand the network characteristics of contagious disease control and the relevant departments in Korea during emergency responses. Disaster-related studies, usually using SNA, have been conducted with social media to analyze the emotions about a particular event [7-9] or analyze certain sections of organizational networks on disaster frameworks [10-12]. The patterns of network formation among every organization related to contagious disease responses were able to be proven, and through this—by identifying the disaster response agencies that play a crucial role in the network structure of response agencies if a substantially sized disaster were to occur—pragmatic policies were provided.

### Korea’s Disaster Response System for Contagious Diseases

**Figure 1:**
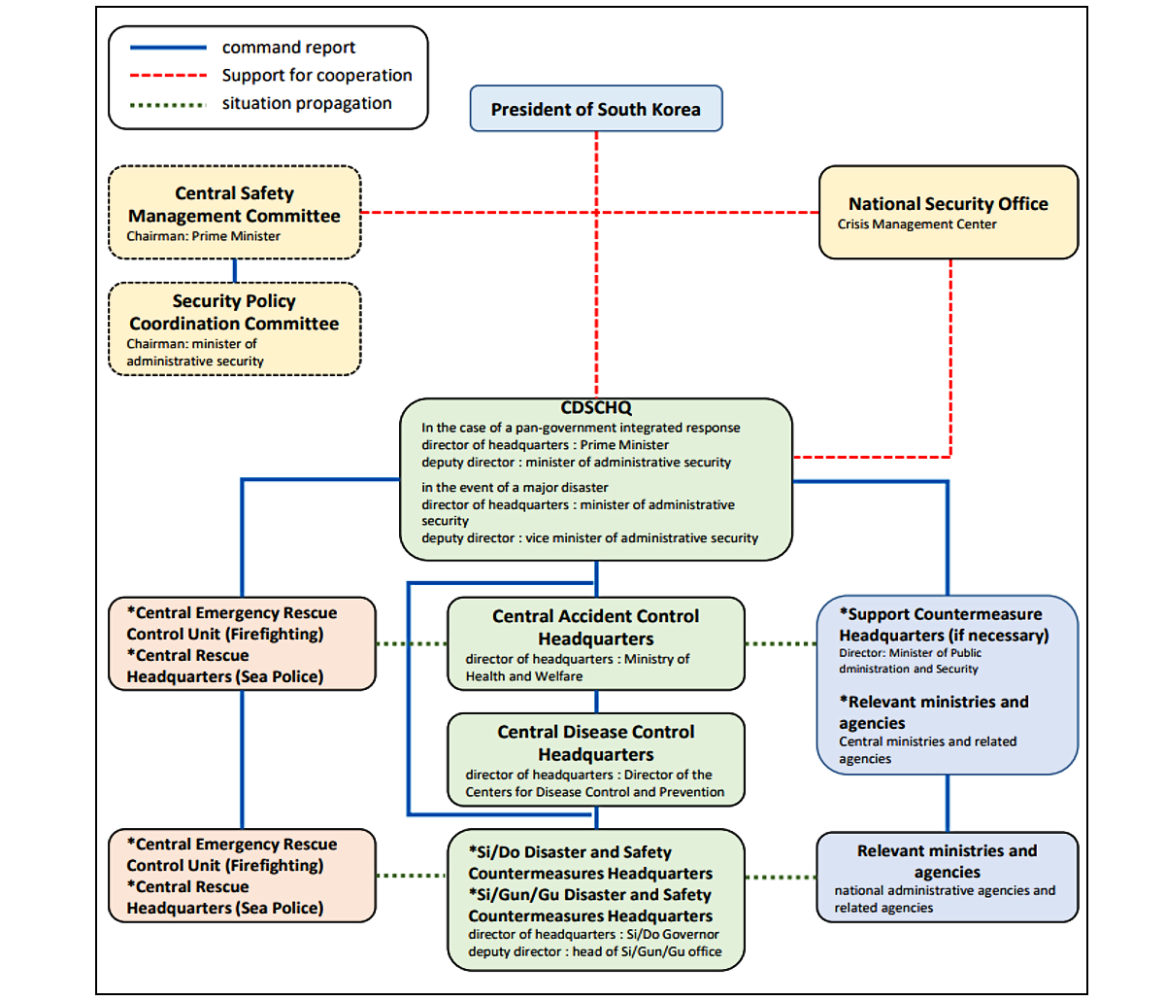
Contagious disease disaster management system. CDSCHQ: Central Disaster and Safety Countermeasures Headquarters; Si/Do: It is an administrative district of the Republic of Korea classified as a metropolitan local government and has a total of 17 'Si' & 'Do'; Si/Gun/Gu: The 'Si' has subordinate administrative districts called 'Gun' and 'Gu,' and the 'Do' has subordinate administrative districts called 'Si' and 'Gu.'.

The constitution of South Korea states that the government consists of a president and its executive branches [13]. At the apex of the hierarchy stands the president, from which orders are given to the Prime Minister who supervises and directs the secretaries of the central administrative agencies. The executive branch consists of 18 departments, 5 offices, 4 offices in 2 houses, 7 committees, and the Deputy Prime Minister (who performs specially delegated affairs), which all fall under the Prime Minister. More often than not, the government—equipped with advice from experts in relevant fields—reaches out to disaster management agencies in the event of a large-scale disaster such as COVID-19. In simpler terms, the Central Disaster and Safety Countermeasures Headquarters (CDSCH) and Central Disaster Management Headquarters (CDMH) are operated by Korea’s disaster response system on a level that is on par with the central government in the case of a national disaster. Figure 1 shows the contagious disease management and response system entailed in the disaster management standard manual. One should take note that the Ministry of Security and Public Administration directs the CDSCH and the CDMH, while the Ministry of Health and Welfare has a central disease management headquarters under its wing to respond to contagious diseases [5].

The Ministry of Health and Welfare, shown in Figure 1, plays a central role in the infectious disease management and response system. Naturally, the CDMH, which falls under the Ministry of Health and Welfare, was also a subject for this study. See Multimedia Appendix 1 for all the institutions and countries included in this study.

## Methods

### Social Network Analysis for COVID-19

#### Concept of Social Network Theory

A social network consists of a web of interpersonal relationships that can be characterized by interactions and interconnections in social relationships [5,14,15]. The actors may be individuals, but the term also refers to entities such as groups, organizations, and companies [16]. The social network theory can be explained through the duality of structure, which is a concept proposed by the British social scientist, Giddens [17]. He defined structure as a medium of action and simultaneously as a product of reproducing an action—determined by the duality of the structure. In essence, in light of the social network theory, the structure of social networks is formed by the actors, and it affects their behavior.

#### Social Network Analysis

It can be said that SNA manages the following: deriving the characteristics of a structure or from the endpoint of a period, explaining a system’s characteristics from a relationship point of view, and the behavior of the units that constitute a system [18]. The main focus of network analysis is identifying the patterns of interactions between the entities making up the network or from the results [19]. Nodes represent actors (eg, people, organizations, groups, events), while the links represent the relationships among the actors. A connection network composed of nodes and lines can be analyzed by grafting them onto social phenomena—hence, SNA [20].

The main approach in SNA is to establish the centrality of the actor where it can be expressed as a value between 0 and 1: 0 means that it is an isolated node without any connection, while 1 means that it is connected to every other node. In other words, the closer the value is to 1, there is greater involvement of a node within a network [21]. The concept of centrality is classified further into “degree of centrality,” “closeness centrality,” and “betweenness centrality.” Degree of centrality simply represents how much one actor is connected to another, which is obtained by adding the total number of connected relationships [22]. Closeness centrality measures the distance between actors within a relationship to identify the network with the most influence [19]. Finally, betweenness centrality measures the extent to which a network is on a path in breaking the flow of information: It sums up the rate of an actor between 2 other actors in the shortest path possible [23].

SNA comprises social units such as events and organizations, as well as information such as the relationships among people [24]. By paying attention to the structure and actions, it can investigate social facts in regards to which agencies have certain relationships and how they are organized.

### Collaboration With Government Agencies in the Event of a Disaster

Not only do contagious diseases such as COVID-19 pose a threat, but various natural disasters—such as wind, floods, and wild fires—occur repeatedly every year, and the scale of damage continues to increase. It is during the times when a large-scale disaster causes calamitous damage that a government-orientated disaster response system is established, and in order for this to be true, a mutual, organic, cooperation system is essential [25]. Moreover, in order to effectively control a disaster response, a network of cooperation consisting of local governments, private organizations, and NGOs hinging on the central government is vital [26,27]. Many studies have been conducted on disaster response systems, and in particular, collaboration among organizations participating in disaster response has been confirmed in light of a network approach [27-29]. An example of this would be from Quarshie and Leuschner [28], where the New Jersey state government interacted with government and NGOs during Hurricane Sandy. As can be seen from the study, the government played a major role in organizing, facilitating, and supplying network members, and it served as the central hub among institutions. A study by Jovita et al [30] analyzed the causes for failing to respond adequately to typhoon Washi, which caused mass destruction to the Philippines in 2010. From the analysis, the networks of each institution participating in the disaster response in the region were very low, which equates to fragile cooperation among the institutions [30].

By analyzing the cooperative system among the government and other related organizations that are involved in a disaster response system, the aforementioned cases confirm the relationship-perspective characteristics and the effectiveness of disaster response systems among the relevant organizations. Thus, the purpose of this paper was to understand the relational characteristics of each institution in a disaster response system.

### Research Design

In order to conduct a proper analysis of social networks, the ranking and roles of responding agencies to COVID-19 were examined to clarify the networks that had been formed to respond to the pandemic (Table 1). When conducting the case study of the organizations, the following criteria were used: First, the agencies included in the contagious disease management and response system suggested in the Korean Disaster Management Standard Manual were the primary focus. Second, agencies that were involved in responding to contagious disease outbreaks were mainly selected. Finally, COVID-19 response was conducted not only among domestic agencies but also with other countries, which amounts to a total of 63 agencies and countries.

This research sought to define relationship aspects among agencies in networks. Therefore, based on the official documents of activities uploaded on the website of the contagious disease disaster response department and the agencies pertinent to it, a node was defined as a contagious disease response organization only if it were noted that a “meeting” was held or “support” or “cooperation” occurred.

This study was conducted using the NetMiner software from CYRAM, a data science group, for efficient data analysis. NetMiner is a professional software that is appropriate for analyzing enormous data [31], and it is able to produce data by applying different methods such as SNA techniques, statistics, data mining, and machine learning.

**Table 1:** Concept of this study.

Designation	Significance
Node	This signifies the agency involved in responding to COVID-19.
Link	This signifies bidirectional communication as part of overall communication, such as via meetings, support, and cooperation among institutions.
Network	This signifies a set of links among agencies, such as meetings, response support, and collaboration for COVID-19 as well as COVID-19 response agencies (nodes).

### Hypotheses

The study intended to determine the degree to which COVID-19 response agencies are centered, assuming that the contagious disease response center (in Korea, the Ministry of Public Administration and Security and the Ministry of Health and Welfare) is more central than the other agencies (Hypothesis 1 [H1]). It was also assumed that the contagious disease response center maintained a closer distance than other agencies and formed a network (Hypothesis 2 [H2]). Finally, the study intended to determine which agencies played a key role among COVID-19 response agencies through their betweenness centrality and also posited that collaboration or information transmission would occur through the contagious disease response center (Hypothesis 3 [H3]). Through this, this study proposes the following 3 hypotheses:

1. H1: The COVID-19 response center will have a high degree of centrality.
2. H2: The COVID-19 response center will have a high closeness centrality.
3. H3: The COVID-19 response center will have a high betweenness centrality.

### Data Collection

The data used in this study were based on the official documents of activities uploaded on the website of the department in charge of responding to contagious diseases and the agencies related to it, which amounted to a total of 11,832 documents. Based on the official documents, it was assumed that a 2-way network was formed between the relevant ministries when preparing for and supporting COVID-19 response measures. The total number of connected networks in this study collected through this method came to 11,909.

The course of the data collection ranges from the date of the first infection in Korea until the time when the number of infected people fell to double digits, which amounted to a total of 102 days, with the various activities confirmed by each ministry. The first period starts from the day of the first infection in Korea until when Korean citizens who were residing in Wuhan moved into temporary residential facilities—from January 20, 2020, until February 18, 2020. The second period is from February 19, 2020, to March 14, 2020. This is when the number of domestic cases surged due to the pseudoreligious group, Shincheonji (SCJ), in Daegu and Gyeongsangbuk province. The final period is when the figures began to fall to double digits—from March 15, 2020, to April 30, 2020. Simply put, the 102 days were categorized into 3 periods, with 2079, 5016, and 4814 links being verified, respectively, for each period in chronological order.

## Results

### Overview

**Figure 2:**
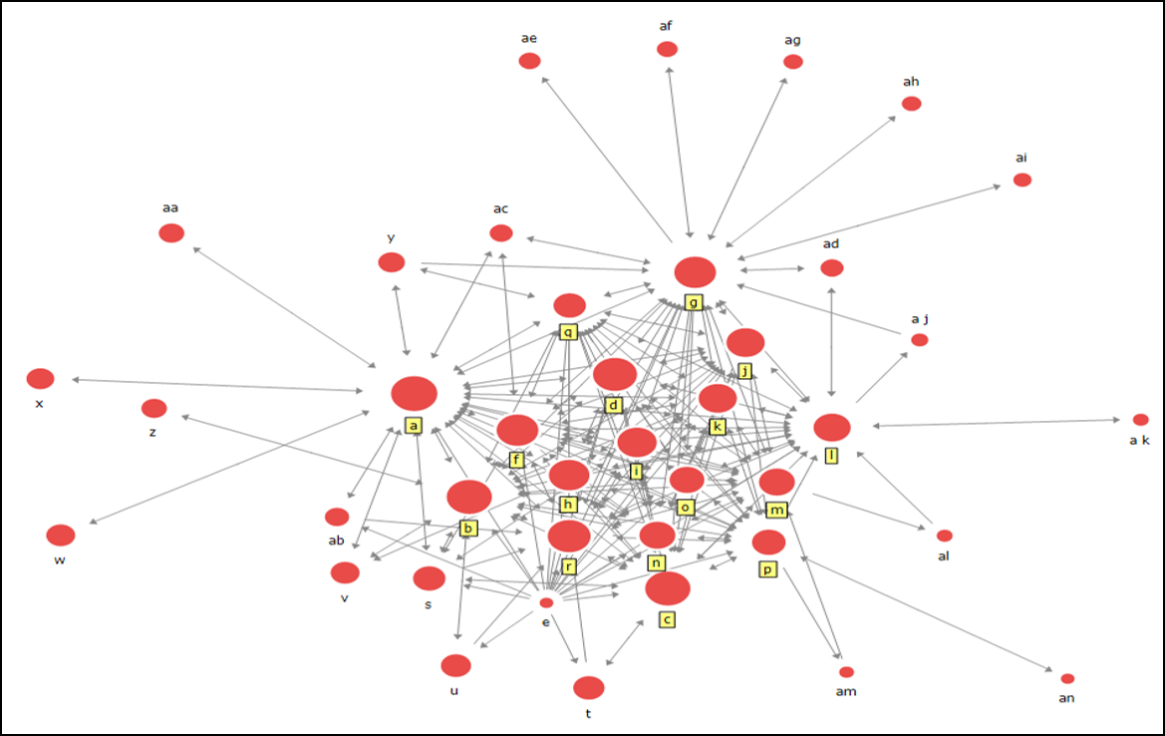
Corresponding management department and associated agencies in a social network with regards to COVID-19 in the first period (January 20, 2020, to February 18, 2020), in which the first case of COVID -19 in Korea was confirmed as well as the transfer of Korean residents from Wuhan to temporary living facilities. The 5 institutions with high centrality are the Korea Disease Control and Prevention Agency, Blue House & President, Prime Minister, Ministry of Economy and Finance, and the Ministry of Oceans and Fisheries.

Figure 2 is a diagram of the social networks of the COVID-19 response department of management and related agencies in the first period. Figure 2 presents the characteristics of social networks that can be identified simply by the node's name. As seen in the corresponding figures, certain institutions have very tight connections. In the diagram, the nodes located in the center and the nodes around it signify centrality, which means that it generally has a higher centrality than other agencies and shows that it plays a key role in the COVID-19 response. A note to take is that, in the first period, the Korea Disease Control and Prevention Agency (KDCA; formally known as Korea Centers for Disease Control and Prevention), which is the department managing the contagious disease response, has the largest node, meaning that it has the most connections with other institutions.

### Centrality During the First Period

The results for degree centrality in the first period are shown in Table 2. In addition to responding to contagious diseases, the Ministry of Strategy and Finance showed the next highest centrality. For that reason, it can be said the Ministry of Economy and Finance is related to agencies responsible for contagious disease responses. During that particular period, events, such as dispatching chartered planes to Wuhan, China, and isolating the infected patients in domestic temporary facilities, occurred. As a result, the Ministry appears to have formed many networks with other agencies as additional revenue had been set aside.

The results for closeness centrality in the first period are shown in Table 3. In-closeness centrality means that the KDCA received the most requests for network formation, maintaining a close distance directly or indirectly to other agencies. Unlike the degree, the Ministry of Food and Drug Safety shows a high out-closeness centrality value. The reason why the Ministry of Food and Drug Safety shows a high out-closeness centrality value is the chaos associated with the regulation that masks be worn to prevent the dissemination of COVID-19. Therefore, in the first period, the KDCA—the management department responsible for responding to contagious diseases—supported H2, since it showed the highest closeness centrality value.

The results for betweenness centrality in the first period are shown in Table 4. The Ministry of Economy and Finance, having shown the highest value in the analysis of betweenness centrality, is the most essential intermediary among other agencies in responding to COVID-19. Following the KDCA, the Ministry of Oceans and Fisheries also showed a high level of betweenness centrality because of previous events such as naval quarantine and the suspension of 16 ports. Judging from these results, the Ministry of Economy and Finance and the related agencies, rather than the department in charge of responding to infectious diseases, showed the highest value in terms of mediated centrality in the first period. Therefore, H3 is not supported.

**Figure 3:**
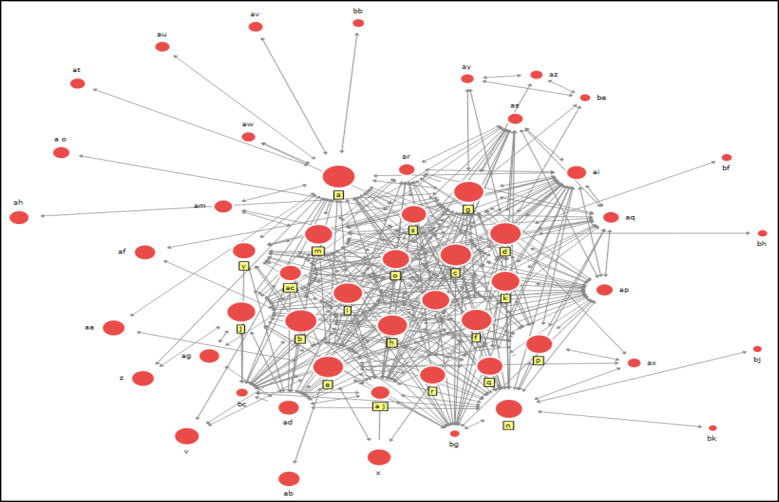
Corresponding management department and associated agencies in a social network with regards to COVID-19 in the second period (February 19, 2020, to March 14, 2020), in which regional infections occurred as the number of confirmed cases surged due to the Shincheonji in Daegu, GyeongBuk Province. The 5 institutions with high centrality are the Korea Disease Control and Prevention Agency, Foreign Ministry, Prime Minister, Ministry of Health and Welfare, and Ministry of Economy and Finance.

Figure 3 is a diagram of the social networks of the department handling COVID-19 responses and the related agencies in the second period, which is also when the largest number of institutions was involved in the COVID-19 response to form a network out of all 3 periods. The number of infected people increased exponentially due to the mass infection that originated from one of the pseudoreligions in Korea—SCJ. SCJ refers to the Korean leader as Jaerim Jesus, and missionary activities are carried out throughout Korea. As a result, confirmed patients at the SCJ Church in Daegu constantly travelled beyond North Gyeongsang Province in Korea to other regions such as Seoul, Gyeonggi Province, and Jeolla Province—dispersing the virus and further heightening the severity of the situation. With this background, the interpretation is that an active network with various institutions was formed to respond to the exponential increase in the number of infected people in the second period.

**Table 2:** Degree centrality of the top 5 agencies in the first period.

Top 5 agencies	Degree centrality	
	In-degree centrality	Out-degree centrality
Korea Disease Control and Prevention Agency (KDCA)	0.619048	0.595238
Ministry of Economy and Finance	0.595238	0.547619
Ministry of Health and Welfare	0.547619	0.52381
The Korean presidential residence (Cheongwadae, the Blue House)	0.52381	0.5
Ministry of Oceans and Fisheries	0.47619	0.452381

**Table 3:** Closeness centrality of the top 5 agencies in the first period.

Top 5 agencies	Closeness centrality	
	In-closeness centrality	Out-closeness centrality
Korea Disease Control and Prevention Agency (KDCA)	0.680233	0.680233
Ministry of Economy and Finance	0.667398	0.655039
Ministry of Health and Welfare	0.643129	0.643129
The Korean presidential residence (Cheongwadae, the Blue House)	0.631645	0.631645
Ministry of Food and Drug Safety	0.631127	0.631127

**Table 4:** Betweenness centrality of the top 5 agencies in the first period.

Top 5 agencies	Betweenness centrality
Ministry of Economy and Finance	0.229265
Korea Disease Control and Prevention Agency (KDCA)	0.190228
Ministry of Oceans and Fisheries	0.098866
Ministry of Health and Welfare	0.089269
The Korean presidential residence (Cheongwadae, the Blue House)	0.082255

### Centrality During the Second Period

The results for degree centrality in the second period are as shown in Table 5. First, when looking at the centrality of internal connections, the 3 highest values came from the Ministry of Foreign Affairs (d), the Ministry of Economy and Finance (g), and Ministry of Health and Welfare (e), respectively.

The results for closeness centrality in the second period are shown in Table 6. The Ministry of Foreign Affairs had received the most requests for network formation, maintaining a close distance directly and indirectly from other agencies. The results indicate that, in the second period, the activities of the Ministry of Foreign Affairs (a related agency), surprisingly not the department of management in charge of responding to infectious diseases, did not support H2 because it showed the highest value in closeness centrality.

The results for betweenness centrality in the second period are shown in Table 7. The Ministry of Foreign Affairs showed the highest betweenness centrality, similar to the degree centrality and closeness centrality. Judging from these results, it can be concluded that the second period did not support H3 because the Ministry of Foreign Affairs showed the highest value in terms of betweenness centrality.

**Figure 4:**
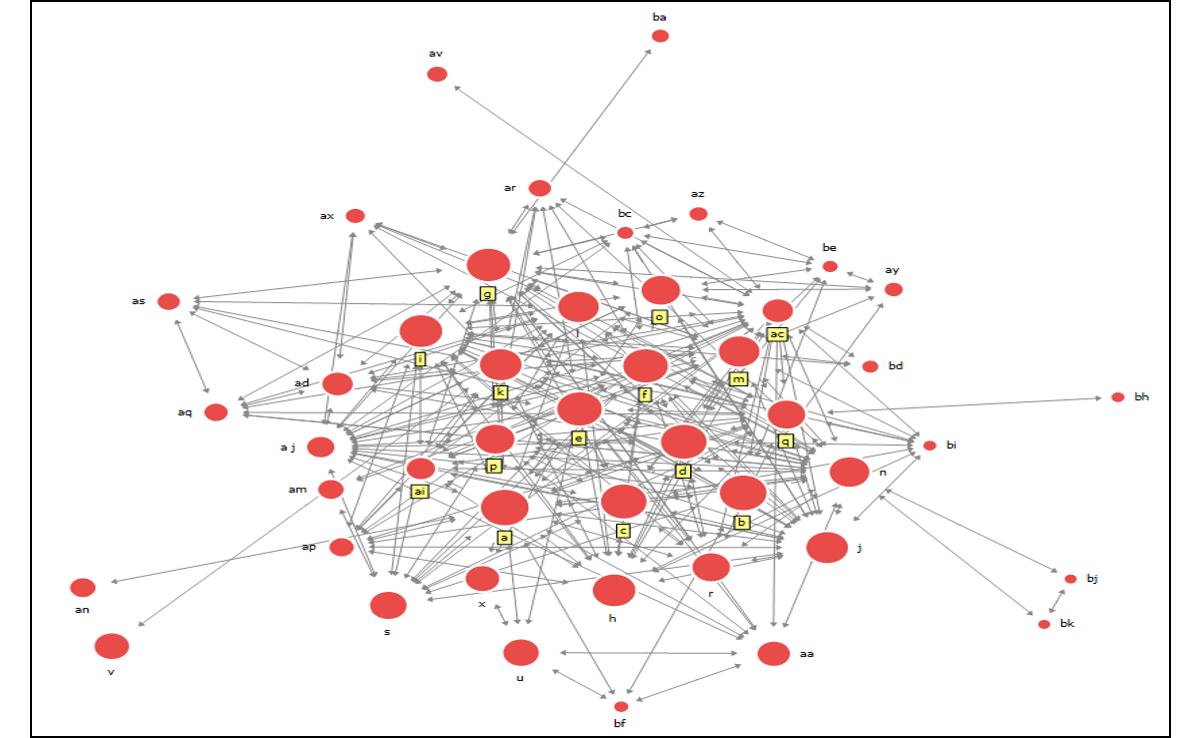
Corresponding management department and associated agencies in a social network with regards to COVID-19 in the third period (March 15, 2020, to April 30, 2020), when the number of confirmed cases began to drop to double digits. The 5 institutions with high centrality are the Foreign Ministry, Ministry of Economy and Finance, Prime Minister, Korea Disease Control and Prevention Agency, and Cheong Wa Dae and President.

Figure 4 is a diagram of the COVID-19 response and management department and its relevant agencies in a social network within the third period. The agency in the center of the network is the Ministry of Foreign Affairs (d), indicated by the largest circle. It is during this period that more than 100 countries enforced restrictions on Koreans for entry, and in the second half of the period, the number of infected people decreased from 3 digits to 2 digits. A repercussion of this was that many overseas countries requested a more robust, international, cooperative system.

**Table 5:** Degree centrality of the top 5 agencies in the second period.

Top 5 agencies	Degree centrality	
	In-degree centrality	Out-degree centrality
Ministry of Foreign Affairs	0.617391	0.6
Ministry of Economy and Finance	0.504348	0.478261
Ministry of Health and Welfare	0.322740	0.313043
Ministry of Science and ICT	0.321739	0.330435
Korea Disease Control and Prevention Agency (KDCA)	0.313043	0.321739

**Table 6:** Closeness centrality of the top 5 agencies in the second period.

Top 5 agencies	Closeness centrality	
	In-closeness centrality	Out-closeness centrality
Ministry of Foreign Affairs	0.656708	0.642809
Ministry of Economy and Finance	0.591037	0.574112
Ministry of Health and Welfare	0.50884	0.498071
Ministry of Science and ICT	0.502188	0.504756
The Korean presidential residence (Cheongwadae, the Blue House)	0.492531	0.485217

**Table 7:** Betweenness centrality of the top 5 agencies in the second period.

Top 5 agencies	Betweenness centrality
Ministry of Foreign Affairs	0.219257
Ministry of Economy and Finance	0.098854
Korea Disease Control and Prevention Agency (KDCA)	0.080244
Ministry of Health and Welfare	0.045238
Ministry of Trade, Industry and Energy	0.032122

### Centrality During the Third Period

The results for degree centrality in the third period are as shown in Table 8. From these results, it is not the department of management responding to contagious diseases, but rather the Ministry of Foreign Affairs (a related organization) that showed the highest value in degree centrality; thus, H1 was not supported.

The results for closeness centrality for this period are shown in Table 9. The Ministry of Foreign Affairs—a related institution—maintained the closest distance to other agencies, and instead of the contagious disease response management department, it showed the highest value in closed centrality, thereby dismissing H2.

The results for betweenness centrality in this period are shown in Table 10.

The Korean presidential residence was the most important intermediary among all other institutions, and this indicates that their activities do not support H3.

**Table 8:** Degree centrality of the top 5 agencies in the third period.

Top 5 agencies	Degree centrality	
	In-degree centrality	Out-degree centrality
Ministry of Foreign Affairs	0.44186	0.44186
The Korean presidential residence (Cheongwadae, the Blue House)	0.364341	0.364341
Ministry of Culture, Sports and Tourism	0.356589	0.356589
Ministry of Trade, Industry and Energy	0.325581	0.325581
Ministry of Health and Welfare	0.310078	0.310078

**Table 9:** Closeness centrality of the top 5 agencies in the third period.

Top 5 agencies	Closeness centrality	
	In-closeness centrality	Out-closeness centrality
Ministry of Foreign Affairs	0.531449	0.531449
The Korean presidential residence (Cheongwadae, the Blue House)	0.519017	0.519017
Ministry of Trade, Industry and Energy	0.513017	0.513017
Ministry of Health and Welfare	0.504272	0.504272
Ministry of Culture, Sports and Tourism	0.495821	0.495821

**Table 10:** Betweenness centrality of the top 5 agencies in the third period.

Top 5 agencies	Betweenness centrality
The Korean presidential residence (Cheongwadae, the Blue House)	0.124024
Ministry of Foreign Affairs	0.094018
Ministry of Trade, Industry and Energy	0.059094
Ministry of Health and Welfare	0.046297
Ministry of Science and ICT	0.044972

### Comparison of Research Results by Period

**Figure 5:**
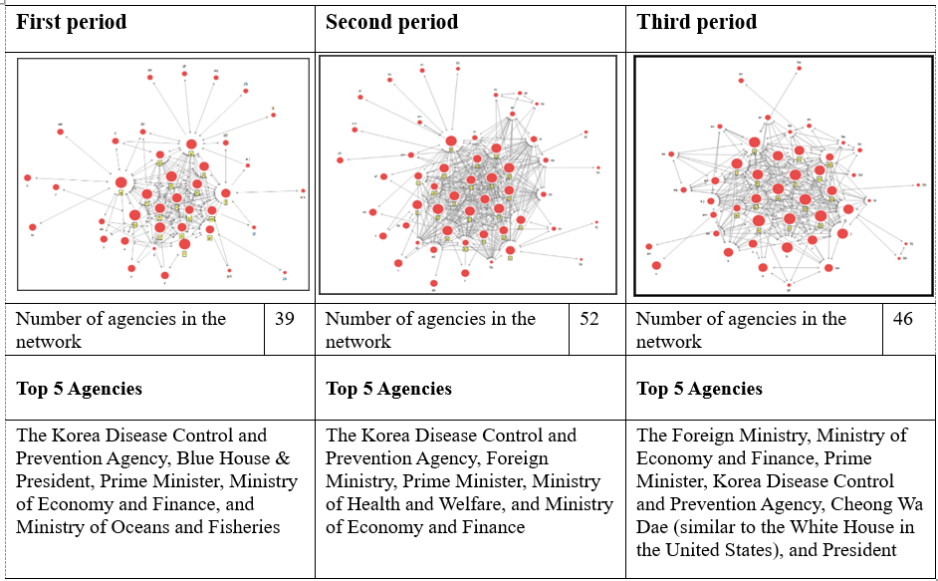
Network diagram comparison of the 3 periods.

Figure 5 shows the results from all 3 periods and the major institutions with a high centrality. The fact that more networks were formed in the second and third periods than in the first period since the COVID-19 outbreak stands out. The explanation for this is that the number of confirmed cases had increased exponentially since the first outbreak in the country, which contributed to the formation of an active network for each institution. In addition, under the Ministry of Health and Welfare, the KDCA had the highest centrality in the first and second periods. However, in the third period, the Ministry of Foreign Affairs—not the center responding to contagious diseases—was located at the center of the network; this can be attributed to 2 factors. First, the role of the Ministry of Foreign Affairs expanded as the number of countries imposing travel restrictions on Koreans rose due to mass infections in Korea at the time, and since then, the number of cases caused by a collective outbreak has decreased sharply. This resulted in the Ministry of Foreign Affairs forming many networks in response to carrying out requests in order to bolster the international cooperative system for the prevention of contagious diseases. In particular, the networks formed in the third period can be said that they show the roles of the host organization in charge of a disaster and that the related organizations are integral to responding to disaster situations.

From the perspective of degree centrality during the 3 periods, the first and second periods showed the highest centrality in the KDCA—a contagious disease agency (Table 11). However, in the third period, the Ministry of Foreign Affairs, an agency related to contagious diseases, showed the highest degree centrality. Degree centrality indicates the degree of information and resource exchange as a frequency linked to other agencies, meaning that the Ministry of Foreign Affairs has conducted many information and resource exchanges with other agencies during the COVID-19 response. Since the values for both in-degree centrality and out-degree centrality are high, this indicates that the desire for other institutions to establish a network with the Ministry of Foreign Affairs is also high, and vice versa.

**Table 11:** Degree centrality of the top 5 agencies for all 3 periods.

Period and top 5 agencies		Degree centrality	
		In-degree centrality	Out-degree centrality
**First**			
	Korea Disease Control and Prevention Agency (KDCA)	0.619048	0.595238
	Ministry of Economy and Finance	0.595238	0.547619
	Ministry of Health and Welfare	0.547619	0.52381
	The Korean presidential residence (Cheongwadae, the Blue House)	0.52381	0.5
	Ministry of Oceans and Fisheries	0.47619	0.452381
**Second**			
	Ministry of Foreign Affairs	0.617391	0.6
	Ministry of Economy and Finance	0.504348	0.478261
	Ministry of Health and Welfare	0.322740	0.313043
	Ministry of Science and ICT	0.321739	0.330435
	Korea Disease Control and Prevention Agency (KDCA)	0.313043	0.321739
**Third**			
	Ministry of Foreign Affairs	0.44186	0.44186
	The Korean presidential residence (Cheongwadae, the Blue House)	0.364341	0.364341
	Ministry of Culture, Sports and Tourism	0.356589	0.356589
	Ministry of Trade, Industry and Energy	0.325581	0.325581
	Ministry of Health and Welfare	0.310078	0.310078

From the perspective of closeness centrality during the 3 periods, it can be confirmed that the result is the same as the aforementioned degree centrality (Table 12). In the case of closeness centrality, the higher the value, the easier it is to reach other organizations in the network, so it usually plays the role of negotiation and coordination. This means that the Ministry of Foreign Affairs oversaw the whole process with other agencies in response to COVID-19 in the second and third periods. In addition, this means that it was able to acquire information in responding to contagious diseases at a faster pace than other institutions.

From the perspective of betweenness centrality during the 3 periods, the Ministry of Economy and Finance had the highest betweenness centrality value in the first period and was the pinnacle agency of all the periods. The Ministry of Foreign Affairs had the highest betweenness centrality value in the second period, while the Blue House ranked first in the third period (Table 13). Organizations with high betweenness centrality have the potential to influence the distribution of information with regards to the control or regulation of information exchange within a network. This happens to be the case since they perform activities that have to do with mediating organizations that do not exchange information on their own. Therefore, the high betweenness centrality value of the related organizations translates to the manisfistation of cooperation among the agencies that support the ones dedicated to responding to contagious diseases. Therefore, those with a high betweenness centrality value (the Ministry of Economy and Finance, the Ministry of Foreign Affairs, and the Blue House) played a mediating role with other agencies because of their position at the core of the network of dedicated agencies.

**Table 12:** Closeness centrality of the top 5 agencies for all 3 periods.

Period and top 5 agencies		Closeness centrality	
		In-closeness centrality	Out-closeness centrality
**First**			
	Korea Disease Control and Prevention Agency (KDCA)	0.680233	0.680233
	Ministry of Economy and Finance	0.667398	0.655039
	Ministry of Health and Welfare	0.643129	0.643129
	The Korean presidential residence (Cheongwadae, the Blue House)	0.631645	0.631645
	Ministry of Food and Drug Safety	0.631127	0.631127
**Second**			
	Ministry of Foreign Affairs	0.656708	0.642809
	Ministry of Economy and Finance	0.591037	0.574112
	Ministry of Health and Welfare	0.50884	0.498071
	Ministry of Science and ICT	0.502188	0.504756
	The Korean presidential residence (Cheongwadae, the Blue House)	0.492531	0.485217
**Third**			
	Ministry of Foreign Affairs	0.531449	0.531449
	The Korean presidential residence (Cheongwadae, the Blue House)	0.519017	0.519017
	Ministry of Trade, Industry and Energy	0.513017	0.513017
	Ministry of Health and Welfare	0.504272	0.504272
	Ministry of Culture, Sports and Tourism	0.495821	0.495821

**Table 13:** Betweenness centrality of the top 5 agencies for all 3 periods.

Period and top 5 agencies		Betweenness centrality
**First**		
	Ministry of Economy and Finance	0.229265
	Korea Disease Control and Prevention Agency (KDCA)	0.190228
	Ministry of Oceans and Fisheries	0.098866
	Ministry of Health and Welfare	0.089269
	The Korean presidential residence (Cheongwadae, the Blue House)	0.082255
**Second**		
	Ministry of Foreign Affairs	0.219257
	Ministry of Economy and Finance	0.098854
	Korea Disease Control and Prevention Agency (KDCA)	0.080244
	Ministry of Health and Welfare	0.045238
	Ministry of Trade, Industry and Energy	0.032122
**Third**		
	The Korean presidential residence (Cheongwadae, the Blue House)	0.124024
	Ministry of Foreign Affairs	0.094018
	Ministry of Trade, Industry and Energy	0.059094
	Ministry of Health and Welfare	0.046297
	Ministry of Science and ICT	0.044972

## Discussion

### Principal Findings

In summary, for 102 days from January 20, 2020, the date of the first infection in Korea, to April 30, 2020, the development of the network of infectious disease response and those of related organizations were categorized into 3 periods, in which this study suggests a few notable ﬁndings: Frst, during the first and second periods, under the Ministry of Health and Welfare, the KDCA had the highest centrality, but in the third period, the Ministry of Foreign Affairs (not the center of the response to contagious diseases) was located at the center of the network. These results show that, in the event of a disaster, not only the leading agency in charge of responding to disasters but also the related agencies are of indusputable importance. Second, regarding closeness centrality, the relationship period, which is not the central agency for responding to contagious diseases, was found to have the highest values of the 2 periods, and looking at betweenness centrality, the related organizations had the highest values in all 3 periods. These results could be an indication of collaboration among related agencies to support dedicated response agencies for contagious diseases for their response. Third, as the hypothesis of this study, the agency dedicated to responding to contagious diseases was expected to have the highest values for all centralities. However, the analysis shows that there are numerous networks formed by related agencies other than the dedicated agencies.

Except in the first period, this study found that the contagious disease response agencies are not situated at the center of the network, which means that they are not in line with the disaster response system created in Korea. In particular, this study’s results show that various institutions are vital for working together to respond to large-scale disasters. In other words, related organizations as well as the host organization should be able to collaborate during a response to a crisis. This means that it is imperative to expand and systemize manuals based on input from institutions that respond to contagious diseases and their related institutions. Therefore, institutional measures are needed to form networks among contagious disease response agencies, and modification of existing disaster response manuals is crucial.

### Limitations

This study has limitations in that its research was contained to only a single type of disaster, COVID-19, and the pandemic has not ended as of the time of writing. In addition, when data were collected for the SNA, only 2-way networks were collected and analyzed, which resulted in the absence of analysis on the direction of each organization's network.

### Conclusion

Based on the COVID-19 situation that led to the declaration of a pandemic, this study conducted an SNA to understand the characteristics of Korea's contagious disease control department and the related agencies from a network perspective. Therefore, to perform an exploratory analysis of the network formation of institutions that responded to COVID-19 in Korea, SNA studies were conducted on the management of contagious disease disaster response and the establishment of a system.

Except for the first period, the other 2 periods showed that contagious disease response agencies were not the center of the network. These findings reveal that not only the host organization but also various organizations should cooperate to respond to disasters. These results are inconsistent with the existing disaster response system. Therefore, not only organizations that are in charge but also the related agencies should be aware of the cooperative function for crisis response in the event of a disaster. In addition, the study is meaningful in that it is an exploratory study on an actual network conducted between the organizer and related agencies in the outbreak of an actual contagious disease.

## Appendix

Multimedia Appendix 1 COVID-19 domestic and international response agencies and organizations.

## Abbreviations

CDMH: Central Disaster Management Headquarters
CDSCH: Central Disaster and Safety Countermeasures Headquarters
KDCA: Korea Disease Control and Prevention Agency
KIAT: Korea Institute for Advancement of Technology
NGO: nongovernmental organization
SCJ: Shincheonji
SNA: social network analysis
