# Supplementary material for: Korea's Response to COVID-19 According to Set Time Frames, With a Focus on the Network Between the Government and Responding Agencies: Social Network Analysis
Source: JMIR Public Health Surveill. 2022 May 23;8(5):e35958. doi: 10.2196/35958 (PMC9128733; doi:10.2196/35958)
Supplement: Multimedia Appendix 1 [file publichealth_v8i5e35958_app1.doc]

Appendix 1. COVID 19 domestic and international response agencies and organizations list &labeling

| Name of agency | Labeling | Name of agency | Labeling |
| --- | --- | --- | --- |
| Korea Centers for Disease Control & Prevention(KCDC) | a | Korea Center for International Finance(KCIF) | ah |
| The Korean presidential residence(Cheongwadae, the Blue House) | b | Ministry of Agriculture, Food and Rural Affairs | ai |
| Prime Minister | c | Ministry of SMEs and Startups | a j |
| Ministry of Foreign Affairs | d | Shipping Company | a k |
| Ministry of Health and Welfare | e | Port Authority | al |
| Ministry of the Interior and Safety | f | Religious World | am |
| Ministry of Economy and Finance | g | Korean Society for Laboratory Medicine | an |
| Ministry of National Defense | h | National Human Rights Commission of Korea | a o |
| Ministry of Education | i | Ministry of Environment | ap |
| Ministry of Justice | j | Korea Customs Service | aq |
| Ministry of Land, Infrastructure and Transport | k | Korean Intellectual Property Office | ar |
| Ministry of Oceans and Fisheries | l | National Tax Service | as |
| Ministry of Culture, Sports and Tourism | m | Ministry of Government Legislation | at |
| Ministry of Trade, Industry and Energy | n | Anti-Corruption & Civil Rights Commission | au |
| Ministry of Employment and Labor | o | Korea Post | av |
| Ministry of Food and Drug Safety | p | Ministry of Patriots and Veterans Affairs | aw |
| Korean National Police Agency | q | Public Procurement Service | ax |
| Korean National Fire Agency | r | Saudi Arabia | ay |
| Local Government | s | The World Bank | az |
| Selective Care Center | t | International Monetary and Financial Committee | ba |
| National Medical Center | u | United States Forces Korea | bb |
| Airport | v | Ministry of Gender Equality and Family | bc |
| Public Health and Environment Research Institute | w | Statistics Korea | bd |
| Health Insurance Review & Assessment Service(HIRA) | x | European Union | be |
| Fair Trade Commission | y | World Health Organization | bf |
| Supreme Prosecutor's Office Republic of Korea | z | Ministry of Unification | bg |
| National Institute of Health | aa | United Nations | bh |
| Public Health Center | ab | Ministry of Personnel Management | bi |
| Ministry of Science and ICT | ac | Korea International Trade Association | bj |
| Finance Service Commission | ad | Korea Trade-Investment Promotion Agency | bk |
| Bank of Korea | af | Korea Center for International Finance(KCIF) | ah |
| Financial Supervisory Service | ag |  |  |
